# Milk-Alkali Syndrome as a Cause of Hypercalcemia in a Gentleman With Acute Kidney Injury and Excessive Antacid Intake

**DOI:** 10.7759/cureus.13056

**Published:** 2021-02-01

**Authors:** Veshesh Patel, Divy Mehra, Brenda Ramirez, Alfredo Lindo, Manuel Suarez

**Affiliations:** 1 Osteopathic Medicine, Nova Southeastern University Dr. Kiran C. Patel College of Osteopathic Medicine, Fort Lauderdale, USA; 2 Ophthalmology, Nova Southeastern University Dr. Kiran C. Patel College of Osteopathic Medicine, Miami, USA; 3 Internal Medicine, Aventura Hospital and Medical Center, Aventura, USA; 4 Pulmonology and Critical Care Medicine, Westchester General Hospital, Miami, USA

**Keywords:** hypercalcemia, parathyroid hormone, metabolic alkalosis, milk-alkali, acute kidney injury

## Abstract

Among the pertinent differentials for hypercalcemia, milk-alkali syndrome remains a diagnosis of exclusion following a thorough workup of other severe causes. However, several key signs may increase a clinician's index of suspicion for possible milk-alkali syndrome, including a prolonged history of antacid ingestion. Milk-alkali syndrome commonly presents with a classic triad: hypercalcemia, metabolic alkalosis, and acute kidney injury. The diagnostic workup should include evaluation of both serum and urine calcium levels, serum phosphate levels, and other hormones (parathyroid hormone, vitamin D). In the case of a confirmed diagnosis of milk-alkali syndrome, rapid correction of calcium levels is of utmost importance. We present the case and workup of an individual presenting to the emergency room with hypercalcemia, acute kidney injury, and several key systemic symptoms. Given a significant history of antacid overuse, and a thorough diagnostic workup, a diagnosis of milk-alkali syndrome was made and the patient was treated accordingly, making a full recovery. We review this rare case and important clinical pearls regarding milk-alkali syndrome.

## Introduction

Milk-alkali syndrome presents with a triad of hypercalcemia, metabolic alkalosis, and acute kidney injury associated with the ingestion of calcium-rich medications and absorbable alkali. There has been an increase in incidence of this rare syndrome, presenting as an increasingly common cause of hypercalcemia [1]. Hypophosphatemia, due to an absence of phosphate load as well as phosphate binding to excess calcium, and hypomagnesemia, due to hypercalcemia inhibiting magnesium reabsorption by the renal tubule, are common at presentation. Intact parathyroid hormone (PTH) levels are usually reduced due to negative feedback at the level of parathyroid glands resulting from hypercalcemia of milk-alkali syndrome [1]. However, making the diagnosis of milk-alkali syndrome requires ruling out other causes of hypercalcemia, such as multiple myeloma, primary/secondary hyperparathyroidism, sarcoidosis, squamous cell carcinoma of the lung [2], vitamin D toxicity, and others. Most diagnostic workups consist of evaluating the serum and urine calcium, serum phosphate, intact PTH, and parathyroid hormone-related peptide (PTHrP) [3]. This case details a unique presentation of a rare syndrome.

## Case presentation

A 70-year-old homeless man with a past medical history of hypertension, history of spontaneous deep vein thrombosis (DVT), a thoracic aorta aneurysm, and treated hepatitis C presents to the emergency room complaining of pain in all four extremities with associated abdominal pain for the past five weeks. He describes the extremity pain as sharp, constant, 9/10 intensity on a 0-10 scale, for which he has been taking aspirin for pain control. He reports the abdominal pain as mid epigastric, waxing and waning, burning, non-radiating, and transiently relieved with antacids. The patient also reports feeling nauseated for the past three days, with one episode of non-bilious non-bloody emesis, and alternating diarrhea and constipation for the past couple of weeks. He also states he has lost 40 pounds in the past six weeks.

On primary survey, his temperature was at 37.1 degrees Celsius with a heart rate of 69 beats per minute and blood pressure of 102/57 mmHg. There were decreased bowel sounds and tenderness in the epigastric region, but no rebound tenderness or fluid wave. Additionally, there was a limited range of motion and weakness in the bilateral arms and legs secondary to bone pain. Clinically, the patient was in acute distress and anxious. Laboratory findings were remarkable for elevated calcium at 15.1 mg/dL, acute kidney injury, and normocytic anemia. Serum phosphorus level was low at 1.8 mg/dL and serum magnesium was low at 1.3 mg/dL. Abdominal computed tomography (CT) scan showed a 5-cm long segment of wall thickening of the distal descending, proximal transverse colon representing diverticulosis, mild rectal wall thickening, and an “apple core” lesion in sigmoid colon. CT angiography showed a 1-cm nodule in the trachea on the right above the carina. First, the patient was given four units of calcitonin per kilogram (four doses), intravenous (IV) zolendronate and aggressive IV hydration with normal saline. The patient was then admitted to the intensive care unit (ICU) for close monitoring.

Next morning, the corrected calcium serum level was 11.7 mg/dL and acute kidney injury was resolved. Thus, zolendronate and calcitonin were discontinued, and IV fluids were continued. Calcium continued to trend downward, transiently becoming mildly decreased then correcting to normal after minimal amounts of repletion with calcium gluconate. The gentleman remained stable throughout. The patient was transferred out of the ICU. Esophagogastroduodenoscopy (EGD) scan showed a 1.5-cm duodenal ulcer secondary to non-steroidal anti-inflammatory drug (NSAID) use and evidence of prior bleeding. The workup for multiple myeloma, which included protein electrophoresis, showed no evidence of a monoclonal protein. The workups for malignancies were negative and PTHrP came back not elevated. Bronchoscopy demonstrated no masses or lesion. The patient successfully recovered and was discharged after ruling out malignant causes of hypercalcemia and was continued with follow-up in the outpatient setting.

## Discussion

**Figure 1:**
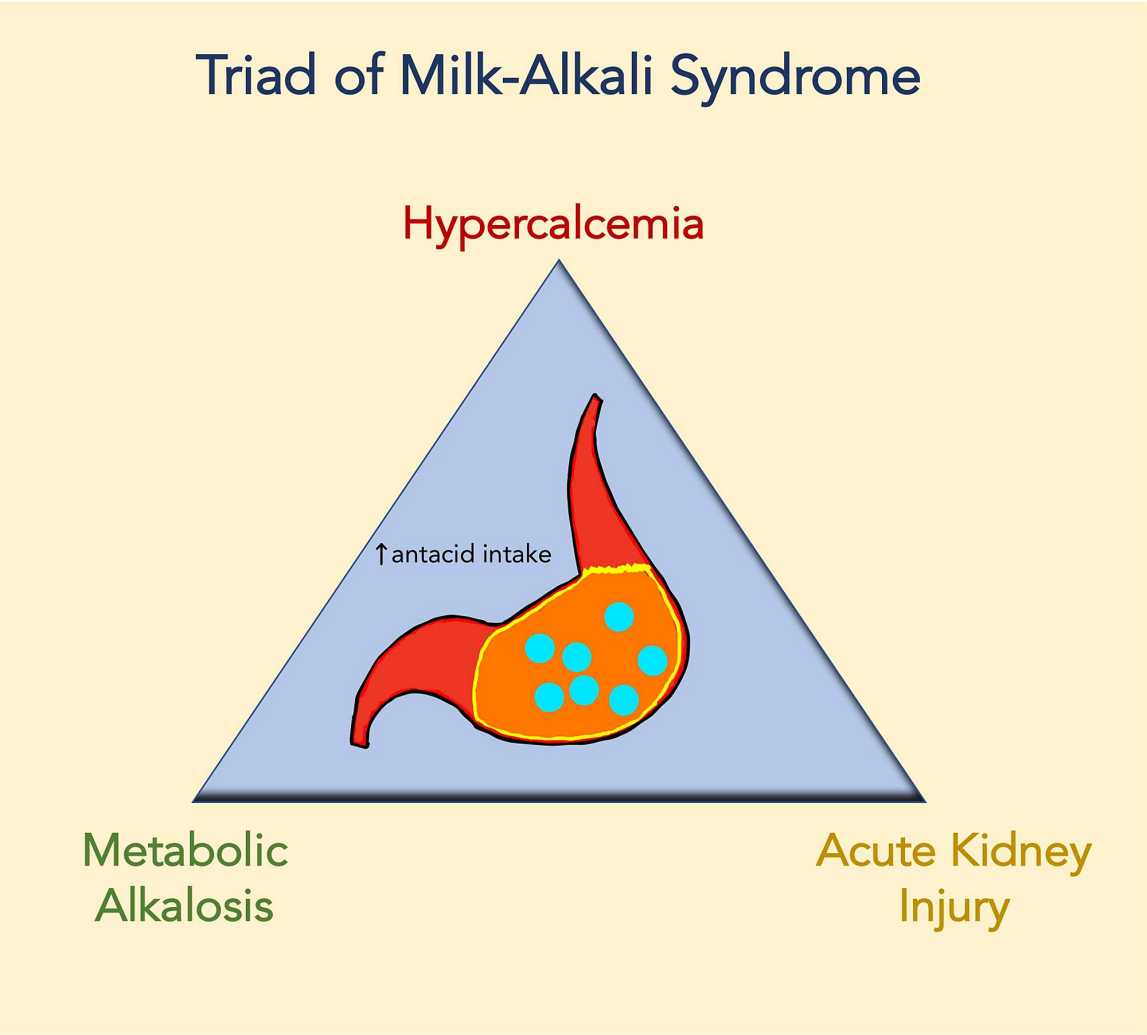
The Triad of Milk-Alkali Syndrome Milk-alkali syndrome commonly presents with a triad of hypercalcemia, metabolic alkalosis, and acute kidney injury, as depicted in the simplified illustration above. A high index of suspicion should be kept for milk-alkali syndrome in individuals with a history of chronic antacid ingestion.

The acute form of milk-alkali syndrome can present commonly with nausea, vomiting, weakness, and mental changes. It is also possible to see severe metabolic alkalosis and acute kidney injury. Treatment typically consists of withdrawal of milk and alkali. The subacute or intermediate form is usually seen in individuals that have been taking excessive milk and alkali intermittently for years (Figure 1). The symptoms are a mix of acute and chronic hypercalcemia. Even with the withdrawal of milk and alkali, kidney function can remain mildly impaired in some cases [4].

A chronic form of the syndrome, known as Burnett’s syndrome, is seen with individuals that have a long history of high milk/alkali intake with symptoms of chronic hypercalcemia such as polyuria, polydipsia, muscle aches, and pruritus. The prognosis is slightly worse than the subacute form. There is minimal improvement in renal function and certain cases can develop chronic kidney disease (CKD) [4,5].

In this case, hypercalcemia resulted in a loss of appetite in the patient which contributed to the weight loss. The syndrome mimicked weight loss similarly seen in cancers and other illnesses [2]. The elevated calcium also contributed to muscle pain and weakness. The patient characterized the extremity pain as waxing and waning depending on when the patient had been taking the antacids. This reasoning also helps to explain the alternating cycles of constipation and diarrhea, as hypercalcemia can affect the gastrointestinal system and smooth muscle-mediated waves of digestion [6]. Stasis and decreased movement in the gut can be a reason for the patient’s wall thickening of the distal descending and proximal transverse colon. These gastrointestinal changes represent diverticulosis, with mild rectal wall thickening and a “core apple” lesion in the sigmoid colon. In the hospital, the patient was confused and agitated, though the vitals were stable and there was no fever. Elevated calcium levels can cause neurological symptoms such as disorientation, difficulty thinking, and depression. The patient’s NSAID use helped to alleviate the extremity pain but contributed to the 1.5-cm duodenal ulcer. For this reason, all the workups for malignancies came back negative.

As exemplified in this case, causes of hypercalcemia can present as a life-threatening condition or a more common benign presentation, mainly depending on the degree of hypercalcemia. Milk-alkali syndrome can mimic a malignancy; laboratory and imaging findings may prove confusing to healthcare providers given a high necessary suspicion for malignancy [2]. Therefore, workups of differential diagnoses are imperative. Timely correction of the elevated calcium, especially when >15 mg/dL, with calcitonin followed with an infusion of isotonic saline, is necessary and may result in rapid clinical improvement. In severe cases, concurrent furosemide therapy can facilitate additional urinary calcium excretion. Suppressed PTH levels are slower to respond - in these cases, administering bisphosphonates can cause a prolonged suppression of the serum calcium, producing hypocalcemia. Our patient had a rapid correction in calcium which made the clinical course more reassuring. The prognosis of milk-alkali syndrome is overall favorable [4].

## Conclusions

Milk-alkali syndrome is an unusual cause of hypercalcemia in individuals presenting with acute kidney injury and has multiple etiological factors. As demonstrated in this case, a high degree of clinical suspicion should be kept for milk-alkali syndrome in individuals with signs of hypercalcemia and a history of excessive antacid digestion. A thorough laboratory and imaging workup is warranted to rule out serious causes, such as malignancies, leaving milk-alkali syndrome to be a pertinent diagnosis of exclusion.
